# Aggressive behavior in patients hospitalised for a psychotic relapse

**DOI:** 10.1192/j.eurpsy.2023.2249

**Published:** 2023-07-19

**Authors:** I. Bouguerra, E. Khelifa, H. Abaza, F. Ben Othman, A. Adouni, H. Ben Ammar, L. Mnif

**Affiliations:** 1 F pyshciatry departement; 2 Razi Hospital, Mannouba, Tunisia

## Abstract

**Introduction:**

Patients in psychotic relapse may exhibit violent behavior towards objects, themselves or others. These behaviors, although usually unconscious, are a common reason for hospitalization and a source of rejection and stigmatization by family and society.

**Objectives:**

The objective of this study was to evaluate the presence of aggressive behavior in relapsed inpatients with schizophrenia in the F psychiatry department at the Razi Hospital in Tunisia.

**Methods:**

This was a descriptive, cross-sectional study of fifty male patients hospitalized for a psychotic relapse who were naïve or discontinuing treatment for at least two months. Patients were assessed using a semi-structured questionnaire and the Overt Aggression Scale (OAS).

**Results:**

The age of the patients included ranged from 17 to 65 years, with an average of 36.4±11.51 years. More than half of the patients were without occupation (58%, N= 29). For personnal history : Seven patients (14%) had attempted suicide ; Eight patients (16%) showed evidence of self-harm ; Thirteen patients (26%) had a history of arrests of which eleven (22%) were incarcerated.The OAS score ranged from to 0 to 35 with a mean at 9.7+/- 10.3. Twenty-seven patients were aggressive (54%).

**Conclusions:**

Preventive strategies should focus more on predicting the aggressive potential of patients with schizophrenia and its socio-professional implication. Perhaps when using a less holistic approach to the disease and when approaching aggressive behavior as a symptom in its own right, we will be able to find other alternative options.

**Disclosure of Interest:**

None Declared

